# Extinct and extant termites reveal the fidelity of behavior fossilization in amber

**DOI:** 10.1073/pnas.2308922121

**Published:** 2024-03-05

**Authors:** Nobuaki Mizumoto, Simon Hellemans, Michael S. Engel, Thomas Bourguignon, Aleš Buček

**Affiliations:** ^a^Evolutionary Genomics Unit, Okinawa Institute of Science and Technology, Okinawa 904-0495, Japan; ^b^Computational Neuroethology Unit, Okinawa Institute of Science and Technology, Okinawa 904-0495, Japan; ^c^Department of Entomology and Plant Pathology, Auburn University, Auburn, AL 36849; ^d^Division of Invertebrate Zoology, American Museum of Natural History, New York, NY 10024-5192; ^e^Institute of Entomology, Biology Centre, Czech Academy of Sciences, České Budějovice CZ-37005, Czech Republic

**Keywords:** actualistic paleontology, collective behavior, fossil record, leadership, movement coordination

## Abstract

Fossils encompassing multiple individuals provide rare direct evidence of behavioral interactions among extinct organisms. However, the fossilization process can alter the spatial relationship between individuals and hinder behavioral reconstruction. Here, we report a Baltic amber inclusion preserving a female–male pair of the extinct termite species *Electrotermes affinis*. The head-to-abdomen contact in the fossilized pair resembles the tandem courtship behavior of extant termites, although their parallel body alignment differs from the linear alignment typical of tandem runs. To solve this inconsistency, we simulated the first stage of amber formation, the immobilization of captured organisms, by exposing living termite tandems to sticky surfaces. We found that the posture of the fossilized pair matches trapped tandems and differs from untrapped tandems. Thus, the fossilized pair likely is a tandem running pair, representing the direct evidence of the mating behavior of extinct termites. Furthermore, by comparing the postures of partners on a sticky surface and in the amber inclusion, we estimated that the male likely performed the leader role in the fossilized tandem. Our results demonstrate that past behavioral interactions can be reconstructed despite the spatial distortion of body poses during fossilization. Our taphonomic approach demonstrates how certain behaviors can be inferred from fossil occurrences.

## Significance

Fossils preserving evidence of animal behavior have rarely been described. Here, we used the postures of two termites preserved in amber to reconstruct their behavior prior to entrapment. Mating termites form so-called tandems, with one following the other. We investigated how tandem behavior is modified on a sticky surface that simulates tree resin, by comparing the trapped tandems with a Baltic amber inclusion containing a female and male termite in spatial orientation resembling a tandem. Our analysis shows that both living trapped pairs and the fossil pair share a characteristic body alignment, suggesting that the fossil pair was in a tandem. Our approach refines the interpretation of fossilized behavior by accounting for spatial signatures left by trapped organisms.

Group-living animals coordinate their movement to stay together while searching for a safe place or feeding site (1). Movement coordination is achieved by adjusting the speed and direction of movements in response to neighbors (2, 3). Such behavioral interactions among group members affect the shape of the whole group (4) and the spatial distribution among neighbors (5). Collective behavior can be observed in a diverse range of organisms, from bacteria to humans (2), indicating the ancient origin of movement coordination. However, evidence of behavioral coordination in the fossil record is rare (6–8). Movement coordination is a dynamic process leading to spatial structures that are often altered during fossilization (9). Although several studies have attempted to infer the behavioral processes employed in the collective behavior of extinct animals (6–8), uncertainty remains in their interpretation.

To evaluate behaviors preserved in the fossil record, it is essential to determine the biotic and abiotic factors that may have influenced the preservation of extinct organisms. Amber, or fossil resin, provides uniquely detailed soft-body preservation with three-dimensional (3D) features ideal for capturing snapshots of “frozen behaviors,” fossils with animals preserved in action (9, 10). The co-occurrence of multiple individuals [i.e., syninclusions, or specifically eusyninclusions (11)] is a valuable source of information for tracking behavioral interactions between extinct organisms, including predator–prey relationships (12–14), mating behaviors (14), and host–parasite associations (15, 16). Nevertheless, the preservation process in tree resin is not instantaneous and induces a range of behavioral responses that interfere with the behavior performed immediately before entrapment. Interaction with fresh sticky resin often results in loss of body parts, defecation, and induction of stress behavior, such as egg laying (17). Thus, to improve the accuracy of inferring behavioral coordination from fossil occurrences, it is essential to identify how the behavior of animals is modified during entrapment and death.

Tandem-running behavior is the simplest movement coordination maintained by leader–follower behavioral interactions (18, 19). In termites, tandem running is performed by a pair of de-winged female and male after the dispersal flight (20). During the mating season, winged termites fly from their natal nests and disperse. After dispersal, both females and males land on the ground or tree trunks and run around searching for a mate. Once encountered, a pair forms a tandem run, in which one follows the other by maintaining close contact with the tip of the leader’s abdomen (21). The follower uses its antennae or mouthparts to maintain contact with the leader. The most important function of the termite tandem run is staying together during nest-site exploration. Behavioral specialization, with distinct leader and follower roles improves stability of the pairing (22, 23). Either females or males or both sexes can play the leader role, depending on the termite species (24). The tandem pairs seek a suitable site to establish their nest and form a lifelong monogamous royal pair.

**Fig. 1.**
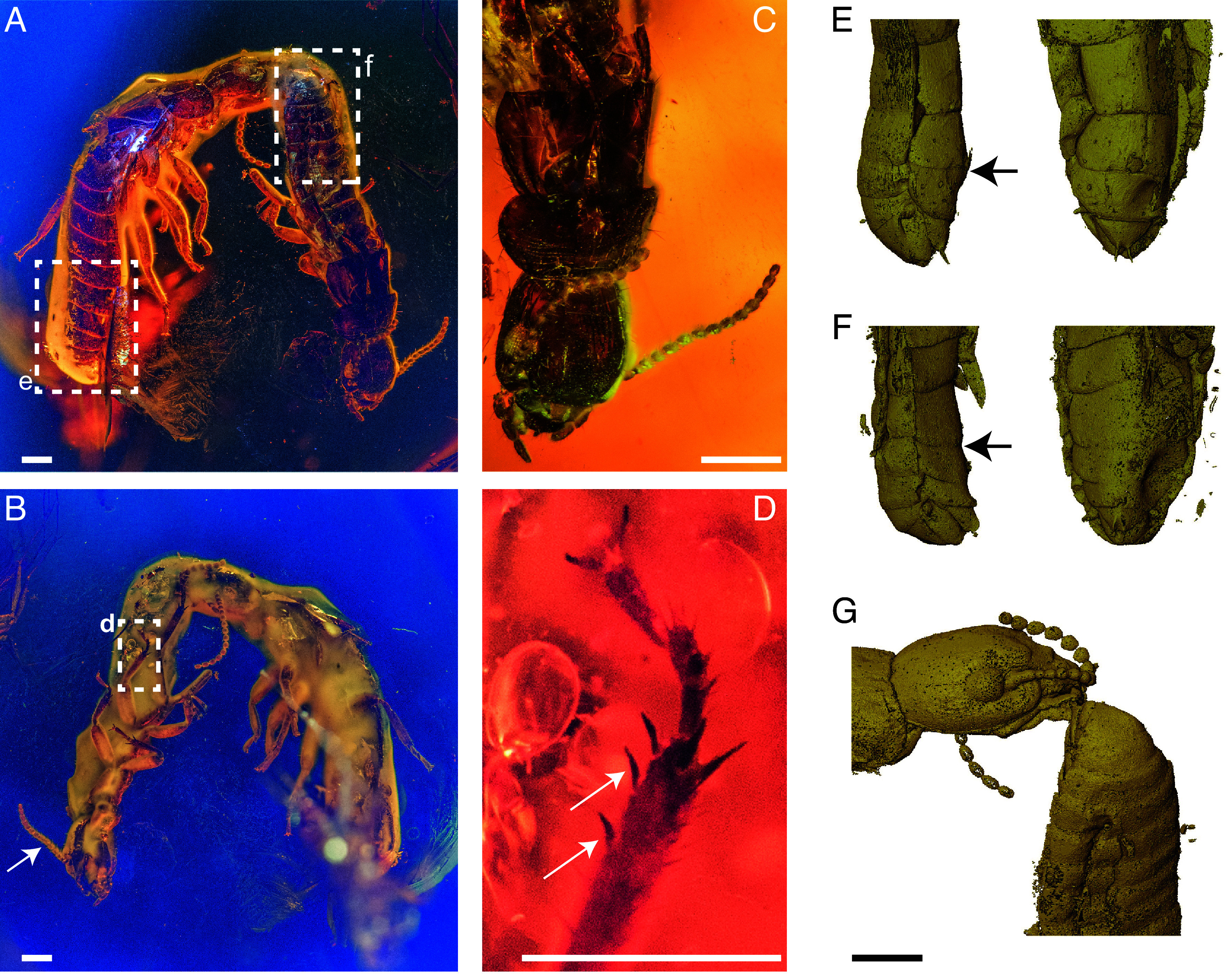
*E. affinis* pair in Baltic amber. (*A* and *B*) The dorsal and ventral sides of the tandem, respectively, with (*B*) an arrow pointing to the 15-articles antenna of the tandem leader. (*C*) Close-up featuring the absence of fontanelle and the pronotum being broader than the head, a combination of features typical of Kalotermitidae. (*D*) Close-up on the middle tibia bearing the two outer spines (see arrows) characteristics of the fossil genus *Electrotermes*. (*E*–*G*) X-ray µCT scans of the abdomen tips of the tandem (*E*) follower and (*F*) leader, with the (*G*) point-of-contact between the follower’s head and the leader’s abdomen. Sternite anatomy (arrows indicate the upper limit of the seventh sternite) revealing that the (*F*) tandem leader is male, and the (*E*) follower is female. (Scale bars are 0.5 mm.)

Eocene Baltic amber is historically the most productive deposit for Cenozoic fossiliferous resin and includes the most diverse fossil insect assemblages (25), including examples of “frozen” behaviors (14) as well as extinct and extant termite genera (26). Termites are abundant in Baltic amber, particularly winged and de-winged termite reproductives. Tandem pairs walking on or near tree trunks (27) are susceptible to entrapment in tree resin, yet fossils of tandem pairs have never been described. Here, we report a unique fossil occurrence of eusyninclusions of two dealate termite imagoes of *Electrotermes affinis* Hagen in 38-My-old Baltic amber (Fig. 1). The eusyninclusions portray a heterosexual composition of the pair, head-to-abdomen orientation, without wings, which are consistent with the two termite individuals representing the occurrence of a fossil tandem pair. However, the side-to-side alignment of the termite pair distinguishes it from the stereotypical front–back alignment in natural tandem runs. We hypothesized that this spatial organization formed during the process of entrapment in the resin and tested this hypothesis by empirically simulating the entrapment process on living termite mating pairs using sticky traps. This taphonomic experiment enabled quantitative measures for more reliably documenting “frozen” behaviors preserved in amber.

## Results

### Amber Eusyninclusions of De-Winged Female and Male Termites.

**Fig. 2.**
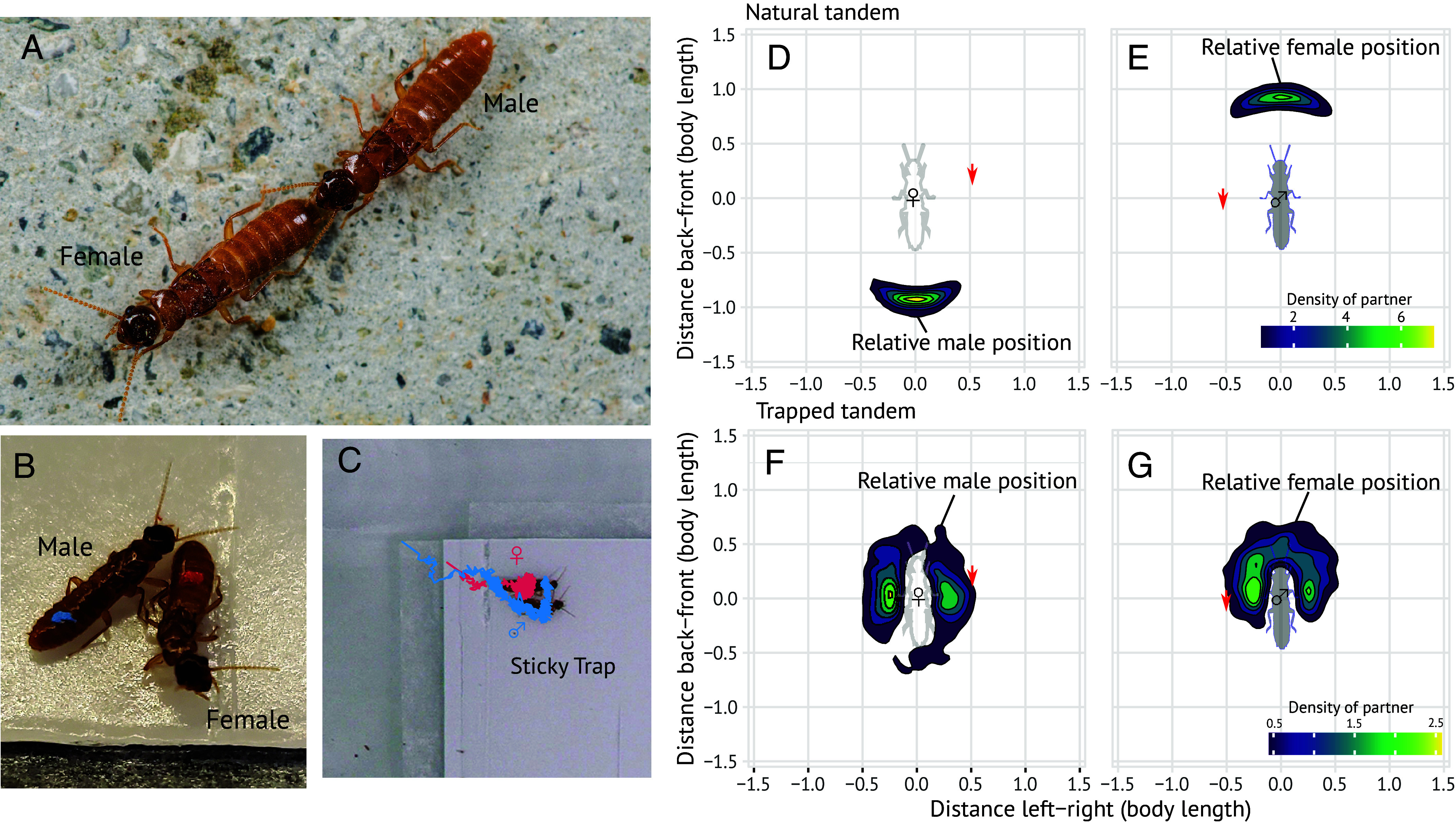
The relative position of females and males forming mating pairs. (*A*–*C*) Mating pairs of the termite *C. formosanus* in (*A*) a natural tandem run and (*B* and *C*) on a sticky surface. Females are marked in red and males in blue. The convoluted lines indicate the trajectories of a female and a male during 30 min after the pair entered the sticky trap. (*D*–*G*) Density map showing the position of one partner, represented by the central shadow of a termite heading towards the top, in relation to the other. The density map was based on pooled data of (*D* and *E*) natural tandem pairs and (*F* and *G*) tandem pairs trapped on the sticky surface. Red arrows indicate the data point of the amber inclusion.

The Baltic amber contains two de-winged drywood termite imagoes (Kalotermitidae) (specimen NMP T3532, Fig. 1 *A*–*C*). The amber consists of multiple layers that are characteristic of successive resin flows (13, 28), and both termites are found in the same flow layer (Movies S1 and S2). Thus, these two termites are eusyninclusions (11), preserved at the same time. An opaque cloud of bubbles visually obscured the ventral posterior parts of the abdomens of the two termites. X-ray microtomography uncovered that one individual was female (Fig. 1 *A* and *E*) and the other was male (Fig. 1 *A* and *F*), based on the anatomy of the 7th sternite, which is enlarged only in females (29). Spines on the mesotibia indicated that both termites belong to the extinct genus *Electrotermes* (30) (Fig. 1 *B* and *D*). Body lengths of ~6.5 mm for the female and ~5.5 mm for the male further confirmed that the species is *E. affinis* rather than the distinctly smaller *Electrotermes girardi* (26, 31). The larger body size of the female imago is consistent with the sexual dimorphism of extant species of Kalotermitidae (32, 33) and further corroborates the sex determinations. The female’s mouthparts were in contact with the tip of the male’s abdomen (Fig. 1*G*), implying that these two termites were performing tandem-running behavior at the moment of entrapment. However, the female and the male within the amber piece were positioned side-by-side (Fig. 1 *A* and *B*), which is unlike living termites in which the leader and follower are in a single file, with the follower just behind the leader (Fig. 2*A*). We hypothesized that the non-instantaneous fixation of trapped termites by sticky tree resin is at the origin of their parallel orientation preserved in the amber.

### Phases of Simulated Entrapment and Movement Dynamics.

To evaluate the effect of the entrapment process on tandem-running behavior, we observed movement patterns of termite mating pairs on a sticky surface. We simulated the tree resin using a sticky trap for insect collection, a method that has been used to mimic the process of resin sampling (34, 35). We then compared the fossil information with the spatial organization of the leader–follower relationships during natural tandem runs (Fig. 2*A*) and after being trapped by sticky traps (Fig. 2*B*).

We introduced a mating pair of the termite *Coptotermes formosanus* Shiraki into an experimental arena with a sticky trap (*SI Appendix*, Fig. S1) and recorded the events during which termite leaders entered the sticky surface (*n* = 26). Although the trap partially obstructed the movement of leaders that naturally entered the sticky traps before the follower, followers did not stop following the leader, resulting in both individuals entering completely, with the whole body area and all appendages onto the sticky surface for all events (Movie S3). On the sticky surface, the movement speed was significantly lower than in natural tandems (sex pooled, Mean ± SD, natural: 1.99 ± 0.51 body-length/sec, sticky: 0.06 ± 0.02 body-length/sec; Wilcoxon rank sum test, *P* < 0.001). Although some individuals escaped from the surface, both the female and male were trapped in 17/26 events after 10 min, 14/26 events after 20 min, and 9/24 events after 30 min. There was no significant difference in the probability of escaping the sticky surface between sexes (mixed effect Cox model, χ^2^_1_ = 0.214, *P* = 0.644). Thus, our sticky trap successfully simulated the situation where termites were not instantaneously immobilized but could behaviorally respond to the entrapment (Fig. 2*C*), potentially resulting in the specific spatial orientation observed in the amber inclusion (Fig. 1).

### Spatial Organization of Trapped Pairs.

The spatial orientation of the leader and the follower after entrapment was significantly different than in natural tandem runs. The distance between the body centroids of the leader and the follower was smaller in trapped pairs than in natural tandems (Fig. 2 *D*–*G* and *SI Appendix*, Fig. S2, Exact Wilcoxon rank sum test, *W* = 599, *P* < 0.001). This is because partners of trapped pairs were often positioned side-by-side, differing from the linear positioning of natural tandems (Fig. 2 *D*–*G*). The shorter inter-individual distance could result from the two individuals entering the sticky surface together and becoming stuck near each other without the ability to move away, rather than their active behavioral interactions to maintain proximity. To test this possibility, we randomly paired trajectories of leader and follower from different trapped pairs. In randomized pairs, the trajectories of the female and male started as if they entered the sticky surface together, but the subsequent progress of each trajectory is independent from each other. We found that the inter-individual distance of these simulated pairs was larger than the real trapped pairs (*SI Appendix*, Fig. S2), indicating that a tandem on a sticky surface stayed together due to active inter-individual interactions. Furthermore, partners within trapped pairs were either heading in the same or opposite direction, which was not observed in randomized pairs (*SI Appendix*, Fig. S3, the distributions of heading direction difference was different between conditions; KS test, *P* < 0.001). Thus, the process of becoming ensnared on a sticky surface modifies the spatial structures of tandem runs in a unique and specifiable way. The observed interindividual distances in the amber inclusion fell well within the range of trapped tandem pairs and outside that of natural tandem runs (Fig. 2 and *SI Appendix*, Fig. S2). Therefore, the observed side-by-side positioning of the two individuals of *E. affinis* in the amber inclusion is expected for an entrapped tandem run.

### Estimating Leader–Follower Roles from Relative Body Postures.

We quantified body postures in trapped and untrapped *C. formosanus* pairs by extracting coordinates of their body parts (head, pronotum, and abdominal tip of each sex). In natural tandem runs, the head of the follower is always adjunct to the abdomen tip of the leader, and the opposite does not happen (Fig. 2*A*). Thus, the distance between female abdomen tip (fTip) and male head (mHead) is much shorter than the distance between female head (fHead) and male abdomen tip (mTip) (*SI Appendix*, Fig. S4*A*). The same pattern was observed in trapped pairs, where fTip–mHead distance was significantly shorter than fHead–mTip distance as in the natural tandems (paired *t* test, *t* = 60.95; *P* < 0.001; *SI Appendix*, Fig. S4*B*). However, the inference of leader–follower roles from head-to-abdominal distances is more ambiguous in trapped tandems than in natural ones. In natural tandems, the distributions of fTip–mHead distance and fHead–mTip distance have no overlaps, while they are overlapped in trapped pairs (*SI Appendix*, Fig. S4). Therefore, although the female head touches the male abdomen tip in the fossilized *E. affinis* pair (Fig. 1 *A*, *B*, and *G*), interpreting the male as a tandem leader is not straightforward.

**Fig. 3.**
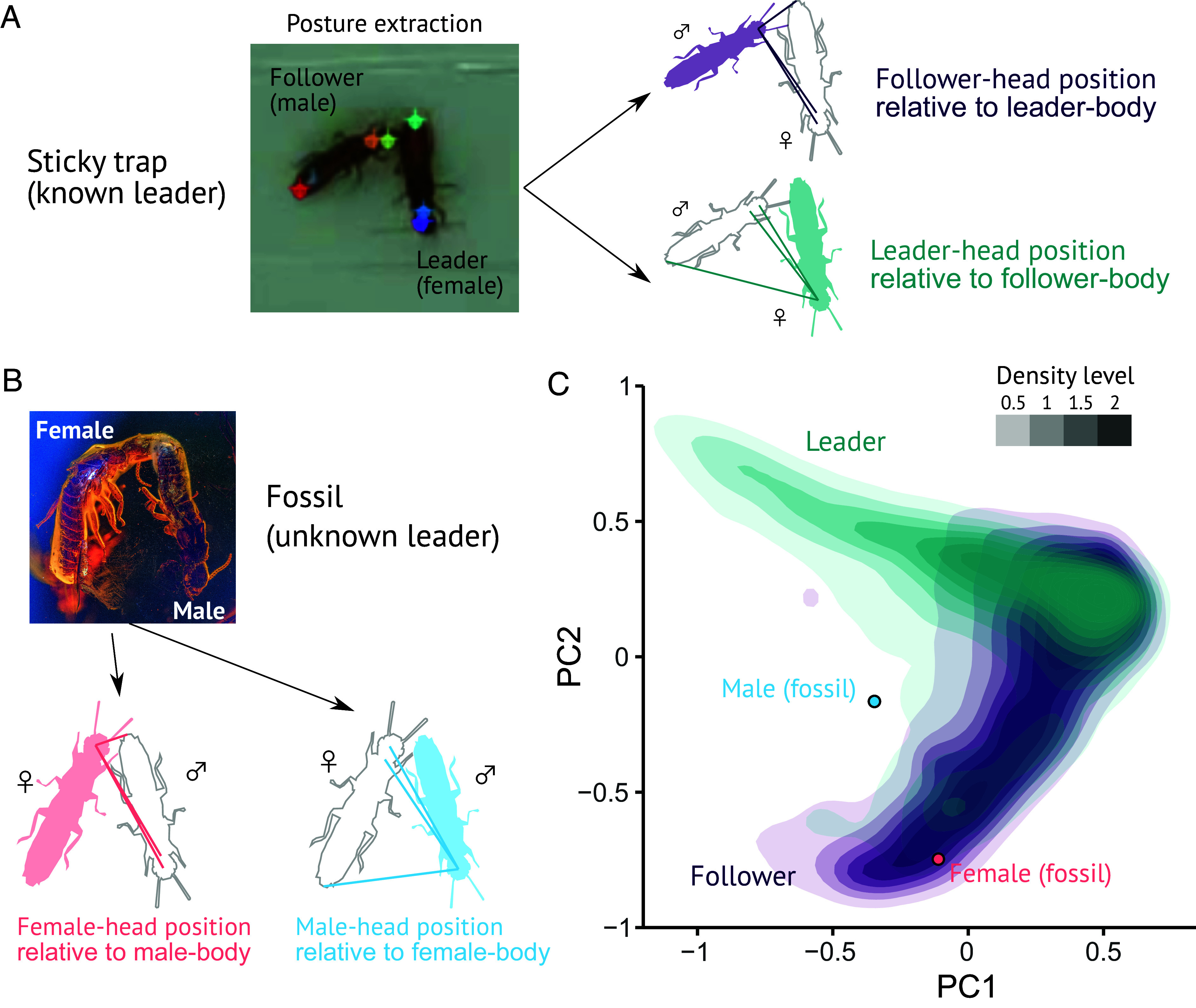
Inference of leader–follower roles from relative postures of termite pairs trapped by sticky surface. (*A* and *B*) Analysis procedure. For each tandem pair, we extracted the body part coordinates (*Middle* of the head, pronotum, and abdomen tip) of both partners. Then, we defined the relative position of one’s head as distances to the three body parts of their partner. (*A*) As the female is always the leader in *C. formosanus* tandems, we labeled females and males as leaders and followers, respectively. (*B*) For the fossilized pair, we obtained the data on the relatives positions of the female and the male. (*C*) Results of the PCA performed on relative positions of leaders and followers of *C. formosanus* and the female and the male of *E. affinis*.

To account for this ambiguity, we quantitatively estimated the probability that the male (or female) was the leader in the amber inclusion pair. To do so, we labeled the females and males of *C. formosanus* as leaders and followers and defined the head positions of leaders and followers using the relative distance to three body parts (head, pronotum, and abdominal tip) of the partner (Fig. 3*A*). We performed a principal component analysis (PCA) on these three distances to observe how the relative head positions of leaders and followers differ (Fig. 3*C*). We further calculated and plotted the relative head positions of the female and the male in the fossilized pair (Fig. 3 *B* and *C*). We found that the relative position of the fossilized female was similar to followers of *C. formosanus* (Fig. 3*C*). Our logistic regression classified the fossilized female as a follower with a probability of 74%. On the other hand, the relative position of the fossilized male deviated from either the leader or follower of *C. formosanus* (Fig. 3*C*). This can be either because the female’s body bent during resin-inclusion process or explained by slightly different body proportions between *E. affinis* and *C. formosanus*. Our logistic regression classified the fossilized male as a leader with a probability of 52%.

## Discussion

Interacting organisms are preserved in amber inclusions in great detail. However, the fixation of organisms in the tree resin is not instantaneous and may alter behavior. Disentangling the natural behavior related to social interactions from the behavioral responses to entrapment is a major challenge in the analysis of behavior fossilized in amber. To solve this problem, we combined a behavior-preserving fossil with taphonomical behavioral experiments that simulate the entrapment process in tree resin. This approach of “actualistic paleontology” (36) provides evidence that a kalotermitid termite, *E. affinis*, performed tandem movement coordination 38 Mya. This interpretation is supported by the ancestral state reconstruction of tandem running behavior in termites, which supported that tandem running behavior was present in the common ancestor of modern termites and has been preserved across kalotermitid species (24). Therefore, the tandem running behavior of *E. affinis* is the oldest termite record of a movement coordination behavior still present across extant relatives. Importantly, our empirical simulations with extant termites indicate that the spatial distribution of the tandem pair partners found in amber is expected to differ from that of untrapped tandems, while still retaining spatial patterns allowing their identification as tandem pairs. Based on the spatial orientation of the interacting termites in amber reported herein, our analyses suggest that the male is more likely the tandem leader in the described fossil tandem. Note that the alternative female-led tandem cannot be rejected in *E. affinis* because the previous ancestral state reconstruction inferred that the common ancestor of extant Kalotermitidae formed both male-led and female-led tandems (24). By simulating the process of living animals being entrapped by sticky objects, our study quantitatively clarified what aspects of behaviors can and cannot be inferred from fossil records.

Some fossils preserve the “frozen” behavior of animals in actions at the moment of death (9, 10). However, our results demonstrate that animals on the sticky trap are not instantaneously immobilized and change their postures on the surface. These experiments imply that the spatial orientation of animals preserved in sticky matrices, such as in tree resin prior to fossilization into amber, is influenced by the process of entrapment. Therefore, the interpretation of fossilized behavior can be dramatically refined or even corrected by observing the behavior of living organisms under entrapment conditions. Some behaviors fossilized in amber may remain unaltered by the entrapment process. For example, the preservation of mating moths *in* *copula* (14) or hell ants grasping prey items (12) suggests that the inter-individual interactions of these behaviors are strong enough not to be disturbed by the movement on the sticky surface. However, entrapment in amber likely affects many other behaviors. For example, insects dispersing through phoresy can be preserved detached from the host insect, perhaps because the host struggled on the sticky surface before complete encasement (37). The consequence of different behavioral responses can be studied using extant relatives. Furthermore, animals have evolved behavioral responses to sticky objects. For example, recent studies have revealed that ants are not passively affected by sticky objects but actively modify them. Red imported fire ants cover sticky surfaces with soil particles to access food resources (38), and granivorous desert ants remove sticky spider webs from nestmates to rescue them (39). Scavenging insects can be attracted by large animals trapped on a sticky surface (11, 35), and the spatial distribution of these insects may have reflected their foraging behavior. Thus, future studies on behavioral responses to sticky objects by animals will increase our understanding of fossil records in amber, as well as shed light on the behavioral capacity of extant insects.

**Fig. 4.**
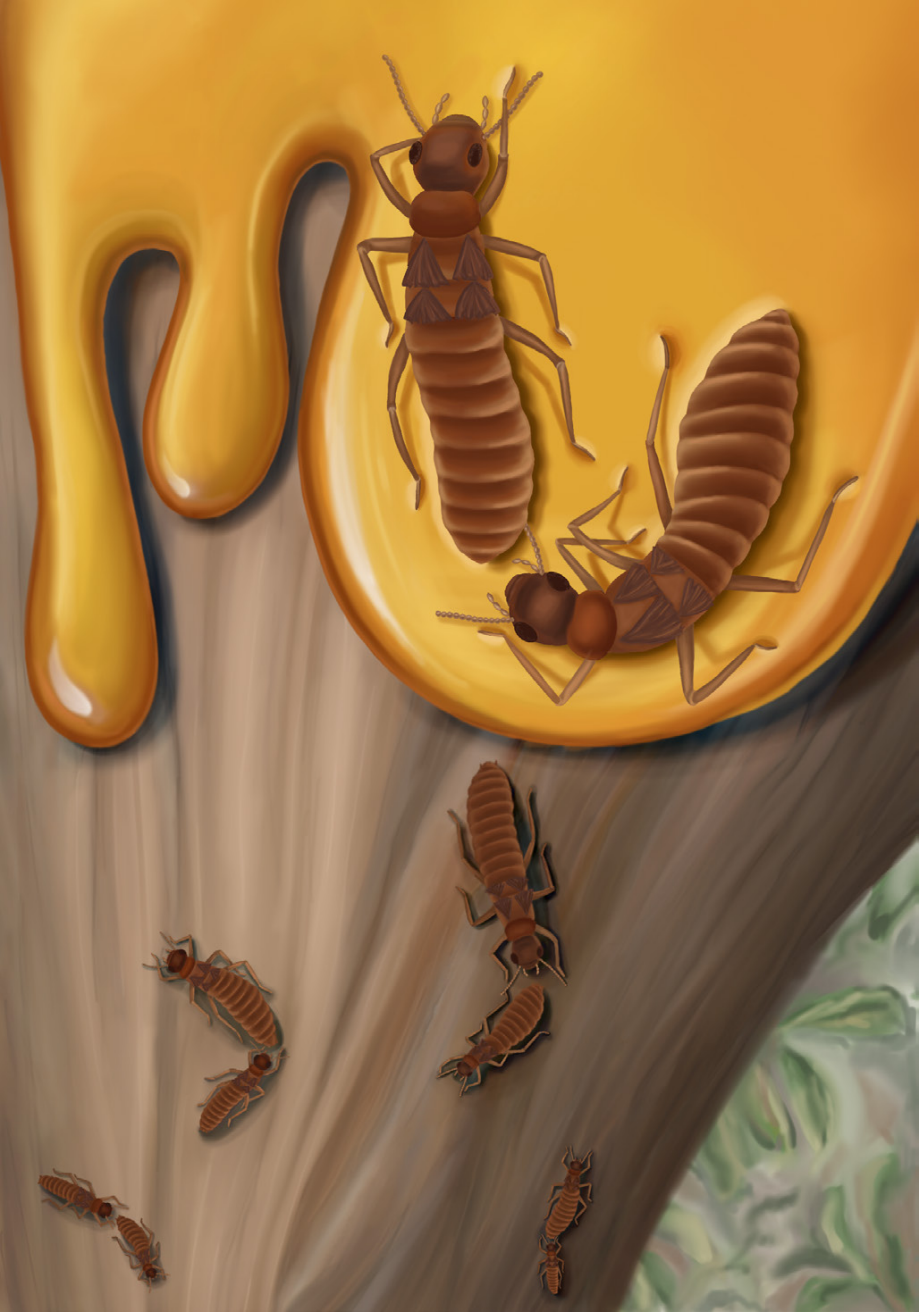
Artistic reconstruction of *E. affinis* tandem pairs running freely on a tree bark and one tandem trapped by tree-resin.

Interestingly, the disturbance of tandem pair movements by a sticky surface is qualitatively distinctive from other sources of disturbance in termite mating pairs. For example, when a tandem pair encounters a predatory ant that captures one of the two partners, an escape behavior is triggered in the other individual (40, 41). When the tandem pair is interrupted by other termites or separated spontaneously, the leader pauses, and the follower moves to search for a partner (23, 27). Hence, termite mating pairs react dynamically to external disturbances in these situations. In contrast, when a sticky trap caught the leader, the follower neither escaped nor left the leader to search for an alternative partner. Instead, the follower walked around the leader and was caught by the sticky trap, resulting in both partners being immobilized side-by-side (Fig. 4). Therefore, sticky surfaces can catch groups of animals, one after another. Social insects, such as ants and termites, are frequently found in groups as amber inclusions (13, 42). Our results show that social interactions of extinct organisms can be inferred from these amber eusyninclusions using a quantitative approach measuring the posture and orientation of fossils in amber, with a comparison of behavioral observations of extant relatives.

In conclusion, we show that spatial data obtained from behavioral observations of animals on a sticky surface are suitable for inferring the posture and relative positioning of animals expressing specific behavior at the time of ensnarement in amber. The behavioral responses of animals to the sticky surface may be slightly different when the sticky surface is not solid but liquid and viscous. The viscosity of resins could be variable depending on the local conditions, age of maturation, and tree species (35). Likewise, the stickiness of the tree resin that formed the amber is unknown. Future studies are needed to investigate the extent to which the different stickiness properties alter the spatial organization of interacting insects. Using an integrative approach to simulate the process of becoming ensnared in resin, fossil records in amber have an untapped potential to uncover the behavior repertoire of extinct animals, especially their social interactions.

## Materials and Methods

### Microtomography of Amber Inclusion.

The amber inclusion containing one female and one male termite originates from the Yantarny mine, Kaliningrad (Russia). It is a polished piece 40 mm long, 23 mm wide, and 7 mm high, and its weight is 4.40 g (see *SI Appendix*, Fig. S5 for overall fossil). The amber fossil was deposited at the National Museum, Prague (Czechia) as specimen NMP T3532. We performed µCT scans of the amber inclusion using a Zeiss Xradia 510 Versa 3D X-ray microscope and the Zeiss Scout-and-Scan Control System software (v11.1.6411.17883). A total of 1,601 projections were collected during 360° rotation of the sample mounted on a rotating carousel. Three scans were performed: two scans of the abdominal tip regions of both termite individuals and one scan covering the region with both termite individuals. For the overview of scanning parameters, see *SI Appendix*, Table S1. The 3D reconstructions of collected projections were performed with the Zeiss Scout-and-Scan Control System Reconstructor software (v11.1.6411.17883). Scan datasets were visualized with the Amira software (v6.7). The voxels representing termite specimens were selected using the “Threshold function,” and subsequently, the outer termite morphology was visualized using the “Volume Rendering” function of the Amira software. Resin flow boundaries were visualized by adjusting the range of values in the “Colormap” module to maximize the visual contrast of the resin flow boundaries. Rotation animation was rendered using the “Animation module.”

### Behavioral Observations.

We used a termite species, *C. formosanus*, as a model organism for observing tandem runs. This extant species shows a typical tandem-running behavior, best described and quantified by the previous studies among other species (19, 21, 23). Also, this species is cosmopolitan and most easily accessible to researchers across the world (43). Although *C. formosanus* belongs to a different family from *Electrotermes*, ancestral state reconstruction suggests that both families inherited their tandem running behavior from their common ancestor (24). Thus, the tandem-running behavior of both species is homologous, and these two species are comparable.

Alates of *C. formosanus* were collected using light trapping in Okinawa, Japan (on the campus of the Okinawa Institute of Science and Technology, near N26.464990, E127.830246) in May and June 2021. After collection, we brought them back to the lab and used individuals that shed their wings for behavioral observations. Individuals were separated by sex and kept on the moistened filter paper until the experiments. All observations were made within 12 h after collection.

We simulated the amber entrapment process using sticky traps for insect collection. Sticky traps have been used to estimate the sampling bias of the insect assemblages captured in amber in tropical forests (34, 35). The processes of entrapment in tree resin have notable differences from entrapment on sticky tapes, such as different adhesive properties and the engulfing properties of fresh tree resin absent in sticky tape. However, arthropod assemblages found in amber or tree resin are similar to those captured by sticky traps (34, 35), suggesting that sticky traps mimic tree resin adequately. We prepared an experimental arena by attaching a trimmed sticky trap (square with 80 mm side; 2-7362-01, ASONE, Japan) to the center of a plastic container (221 × 114 × 37 mm) with double-sided tape (*SI Appendix*, Fig. S1). The surface of the plastic container was roughened so that termites could walk on the surface without slipping. We introduced a tandem-running female–male pair of termites in the arena. The pair was covered by a Petri dish (Φ = 40 mm) until both partners restarted the tandem run (*SI Appendix*, Fig. S1). We recorded the behavior of every pair for at least 20 min after they entered the sticky trap with a video camera (HC-X1500-K, Panasonic) at 30 frames per second (FPS). We stopped observations for pairs that did not enter the sticky trap for 180 min (*n* = 8). Some pairs entered the trap and successfully escaped from the trap. In this case, we resumed the observation until they re-entered the trap. In total, we obtained 26 trap-entering events from 22 pairs. We compared the probability of escaping the trap between sexes, using the mixed-effects Cox model [coxme() function implemented in the R package coxme (44)], with each pair ID included as a random effect.

We used the dataset of natural tandem runs in *C. formosanus* generated by ref. 45 as a control. The experiments were performed using individuals collected on the same day and in the same manner as for the entrapment experiment. The experimental arenas consisted of a petri dish (Φ = 90 mm) filled with moistened plaster. The surface of the arena was cleaned by rubbing off a thin layer of plaster before each trial. Arenas were placed in an acrylic cube box (200 mm) over which a Raspberry Pi Camera Module was mounted on the top board. Camera modules were connected to a Raspberry Pi 4 Computer Model B, and video was recorded using RPi-Cam-Web-Interface (https://elinux.org/RPi-Cam-Web-Interface) at 25 FPS. Thirty-minute video recordings from 27 pairs were available.

### Spatial Organization.

We extracted the coordinates (body centroid) and estimated the heading direction of termites from all videos using the video-tracking system UMATracker (46) (See *SI Appendix*, Fig. S6 for all trajectories). We downsampled all videos to 1 FPS for the downstream analyses. From the data of coordinates and heading directions, we calculated the distance between a female and a male (in body length) and the position of each partner relative to the heading direction of its focal partner (relative direction, [0, π]). The relative direction ranged from 0 (the partner was just in front of the focal individual) to π (the partner was just behind the focal individual). For these analyses, we only used a subset of video frames in which a female and a male were within a distance of twice a termite body length (calculated by summing up female and male body length for each video). This is a conservative threshold for the maximum distance at which two termites forming a tandem can interact (19).

To ensure that the spatial organization observed on sticky surfaces results from inter-individual interactions between partners and not artifacts that may be due to immobilization, we compared the parameters measured from the experimental datasets with randomized datasets. Randomized datasets were artificially created by randomly pairing coordinates of females and males belonging to different tandem pairs after adjusting initial coordinates and the heading direction of swapped males to that of the original males. This randomization process breaks possible behavioral interactions within the randomized pair, while maintaining the influence of a sticky surface on both females and males from different pairs (19). We repeated this process 1,000 times, obtaining 1,000 randomized datasets. We measured the same parameters as above to compare the results with the original datasets.

### Posture Analysis.

We used DeepLabCut (version 2.2.1.1) for body part tracking (47, 48). Specifically, we labeled 194 frames taken from 25 videos (95% of which was used for training). We used a ResNet-50 (49, 50) neural network with default parameters for 750,000 training iterations. We used one shuffle for validation and found that the test error was 2.37 pixels, train: 1.07 pixels (image size was 164 to 640 by 164 to 412 pixels). We used a p-cutoff of 0.9 to condition the X–Y coordinates for downstream analyses. This network was then used to analyze videos. We tracked six points for each tandem run: female head, female pronotum, female abdomen, male head, male pronotum, and male abdomen, where the head was consistently labeled in the middle, the pronotum at the head–pronotum border, and the abdomen at the tip. We did not use multi-animal DeepLabCut (51) but explicitly labeled female and male body parts separately.

Suppose the postures of the pairs on the sticky surface retain specific spatial properties reflecting their leader–follower role. In that case, one could infer the leader–follower role from the snapshot observed in the amber inclusion. To investigate this possibility, we labeled the females and males of *C. formosanus* as leaders and followers. We examined the spatial properties of the female and the male of the fossilized *E. affinis* pair to infer their roles as leader or follower. We computed the relative position of the leaders and followers using the relative distance between their head and three body parts of their partners (head, pronotum, and abdominal tip) (Fig. 3*A*). We performed a PCA on these three distances, where the first two components explained 99.8% of the variability (PC1: 54.5%, PC2: 45.3%). PC1 was negatively correlated to all three distances, indicating that this represents the overall distance between partners (loading; head–head: −0.68, head–pronotum: −0.67, head–abdomen: −0.28), while PC2 represents the distance between head and abdomen (loading; head–head: −0.26, head–pronotum: −0.13, head–abdomen: 0.96). Finally, we performed a logistic regression on these three distances to classify the positions of the two fossilized termite specimens, using the data of *C. formosanus* as a training dataset (52). All data analyses were performed using R v. 4.0.1 (53).

## Supplementary Material

Appendix 01 (PDF)

Movie S1. Rotating view of a reconstructed microtomographic section of the fossil amber piece. Termites are highlighted in orange. The amber matrix is visualized as a volume using a range of grey intensities to distinguish subtle differences in material densities. Resin flow boundaries are visible as faint undulating planes.

Movie S2. Animated slicing view through the reconstructed microtomographic section of the fossil amber piece. The area corresponding to the termites is highlighted in orange on each slice. The amber matrix is visualized as a volume using a range of grey intensities to distinguish subtle differences in material densities. Resin flow boundaries are visible as faint undulating lines.

Movie S3. Example of a termite mating pair caught by a sticky trap.

## Data Availability

All movement trajectories and source codes for analyzing them are available at Github: https://github.com/nobuaki-mzmt/tandem-fossil (54). µCT data in DICOM (digital imaging and communications in medicine) format and codes are available at Zenodo https://doi.org/10.5281/zenodo.10557251 (55). The amber fossil was deposited at the National Museum, Prague (Czechia), as specimen NMP T3532.

## References

[1] I. D. Couzin, Collective animal migration. Curr. Biol. **28**, R976–R980 (2018). 30205074 10.1016/j.cub.2018.04.044

[2] D. J. T. Sumpter, The principles of collective animal behaviour. Philos. Trans. R Soc. B: Biol. Sci. **361**, 5–22 (2006). 10.1098/rstb.2005.1733 PMC1626537 16553306

[3] J. E. Herbert-Read, Understanding how animal groups achieve coordinated movement. J. Exp. Biol. **219**, 2971–2983 (2016). 27707862 10.1242/jeb.129411 PMC5091654

[4] C. K. Hemelrijk, H. Hildenbrandt, Schools of fish and flocks of birds: Their shape and internal structure by self-organization. Interface Focus **2**, 726–737 (2012). 24312726 10.1098/rsfs.2012.0025 PMC3499122

[5] M. Ballerini , Interaction ruling animal collective behavior depends on topological rather than metric distance: Evidence from a field study. Proc. Natl. Acad. Sci. U.S.A. **105**, 1232–1237 (2008). 18227508 10.1073/pnas.0711437105 PMC2234121

[6] N. Mizumoto, S. Miyata, S. C. Pratt, Inferring collective behaviour from a fossilized fish shoal. Proc. R. Soc. B: Biol. Sci. **286**, 20190891 (2019). 10.1098/rspb.2019.0891 PMC6545072 31138077

[7] J. Vannier , Collective behaviour in 480-million-year-old trilobite arthropods from Morocco. Sci. Rep. **9**, 1–10 (2019). 31624280 10.1038/s41598-019-51012-3 PMC6797724

[8] X. G. Hou, D. J. Siveter, R. J. Aldridge, D. J. Siveter, Collective behavior in an early Cambrian arthropod. Science **322**, 224 (2008). 18845748 10.1126/science.1162794

[9] A. Boucot, Evolutionary Paleobiology of Behaviour and Coevolution (Elsevier, 1996), 10.1016/B978-0-444-88034-5.50013-3 (8 October 2018).

[10] S. Hsieh, R. E. Plotnick, The representation of animal behaviour in the fossil record. Anim. Behav. **169**, 65–80 (2020).

[11] M. M. Solórzano-Kraemer , Necrophagy by insects in *Oculudentavis* and other lizard body fossils preserved in Cretaceous amber. Sci. Rep. **13**, 2907 (2023). 36808156 10.1038/s41598-023-29612-x PMC9938861

[12] P. Barden, V. Perrichot, B. Wang, Specialized predation drives aberrant morphological integration and diversity in the earliest ants. Curr. Biol. **30**, 3818–3824.e4 (2020). 32763171 10.1016/j.cub.2020.06.106

[13] D. Coty , The first ant-termite syninclusion in amber with CT-scan analysis of taphonomy. PLoS One **9**, 1–10 (2014). 10.1371/journal.pone.0104410 PMC4139309 25140873

[14] T. C. Fischer, M. K. Hörnig, Mating moths (Tineidae, Ditrysia, Lepidoptera) preserved as frozen behavior inclusion in baltic amber (Eocene). Palaeontol. Electron. **22**, 1–10 (2019).

[15] J. A. Dunlop, J. Kontschán, D. E. Walter, V. Perrichot, An ant-associated mesostigmatid mite in Baltic amber. Biol. Lett. **10**, 20140531 (2014). 25209198 10.1098/rsbl.2014.0531 PMC4190962

[16] R.-X. Jiang , Further evidence of Cretaceous termitophily: Description of new termite hosts of the trichopseniine *Cretotrichopsenius* (Coleoptera: Staphylinidae), with emendations to the classification of lower termites (Isoptera). Palaeoentomology **4**, 374–389 (2021).

[17] A. Arillo, Paleoethology: Fossilized behaviours in amber. Geol. Acta **5**, 159–166 (2007).

[18] N. R. Franks, T. O. Richardson, Teaching in tandem-running ants. Nature **439**, 153 (2006). 16407943 10.1038/439153a

[19] G. Valentini, N. Mizumoto, S. C. Pratt, T. P. Pavlic, S. I. Walker, Revealing the structure of information flows discriminates similar animal social behaviors. Elife **9**, e55395 (2020). 32730203 10.7554/eLife.55395 PMC7392607

[20] W. L. Nutting, “Flight and colony foundation” in Biology of Termites, K. Krishna, F. M. Weesner, Eds. (Academic Press, 1969), pp. 233–282.

[21] A. K. Raina, J. M. Bland, J. C. Dickens, Y. I. Park, B. Hollister, Premating behavior of dealates of the Formosan subterranean termite and evidence for the presence of a contact sex pheromone. J. Insect Behav. **16**, 233–245 (2003).

[22] N. Mizumoto, S. B. Lee, G. Valentini, T. Chouvenc, S. C. Pratt, Coordination of movement via complementary interactions of leaders and followers in termite mating pairs. Proc. R. Soc. B: Biol. Sci. **288**, 20210998 (2021). 10.1098/rspb.2021.0998 PMC8277464 34255998

[23] N. Mizumoto, S. Dobata, Adaptive switch to sexually dimorphic movements by partner-seeking termites. Sci. Adv. **5**, eaau6108 (2019). 31223644 10.1126/sciadv.aau6108 PMC6584256

[24] N. Mizumoto, T. Bourguignon, N. W. Bailey, Ancestral sex-role plasticity facilitates the evolution of same-sex sexual behavior. Proc. Natl. Acad. Sci. U.S.A. **119**, e2212401119 (2022). 36346843 10.1073/pnas.2212401119 PMC9674213

[25] D. Grimaldi, M. S. Engel, Evolution of the Insects (Cambridge University Press, 2005).

[26] M. S. Engel, D. A. Grimaldi, K. Krishna, A synopsis of Baltic amber termites (Isoptera). Stuttg. Beitr. Naturkund. B (Geol. Paläontol.) **372**, 1–20 (2007).

[27] N. Mizumoto, A. Rizo, S. C. Pratt, T. Chouvenc, Termite males enhance mating encounters by changing speed according to density. J. Anim. Ecol. **89**, 2542–2552 (2020). 32799344 10.1111/1365-2656.13320

[28] X. Martínez-Delclòs, D. E. G. Briggs, E. Peñalver, Taphonomy of insects in carbonates and amber. Palaeogeogr. Palaeoclimatol. Palaeoecol. **203**, 19–64 (2004).

[29] F. M. Weesner, “External anatomy” in Biology of Termites, K. Krishna, F. M. Weesner, Eds. (Academic Press, 1969), pp. 19–47.

[30] K. Krishna, A generic revision and phylogenetic study of the family Kalotermitidae (Isoptera). Bull. Am. Mus. Nat. Hist. **122**, 303–408 (1961).

[31] H. Weidner, Die Bernstein-Termiten der Sammlung des Geologischen Staatsinstituts Hamburg. Mitteilungen aus dem Geologischen Staatsinstitut in Hamburg **24**, 55–74 (1955).

[32] M. Afzal, Metabolic rates in different castes and instars of a drywood termite *Bifiditermes beesoni* (Gardner) (Isoptera). Int. J. Trop. Insect Sci. **5**, 13–17 (1984).

[33] Y. Miyaguni, A. Agarie, K. Sugio, K. Tsuji, K. Kobayashi, Caste development and sex ratio of the Ryukyu drywood termite *Neotermes sugioi* and its potential mechanisms. Sci. Rep. **11**, 15037 (2021). 34294796 10.1038/s41598-021-94505-w PMC8298410

[34] M. M. Solórzano Kraemer, A. S. Kraemer, F. Stebner, D. J. Bickel, J. Rust, Entrapment bias of arthropods in Miocene amber revealed by trapping experiments in a tropical forest in Chiapas, Mexico. PLoS One **10**, 1–24 (2015). 10.1371/journal.pone.0118820 PMC4364730 25785584

[35] M. M. Solórzano Kraemer , Arthropods in modern resins reveal if amber accurately recorded forest arthropod communities. Proc. Natl. Acad. Sci. U.S.A. **115**, 6739–6744 (2018). 29735653 10.1073/pnas.1802138115 PMC6042089

[36] J. E. Warme, “Actualistic paleontology” in Paleontology, Encyclopedia of Earth Science, C. W. Finkl, Ed. (Kluwer Academic Publishers, 1979), pp. 4–10.

[37] N. Robin, C. D’Haese, P. Barden, Fossil amber reveals springtails’ longstanding dispersal by social insects. BMC Evol. Biol. **19**, 1–12 (2019). 31752661 10.1186/s12862-019-1529-6 PMC6869205

[38] C. Wen , Red imported fire ants (Hymenoptera: Formicidae) cover inaccessible surfaces with particles to facilitate food search and transportation. Insect Sci. **28**, 1816–1828 (2021). 33247536 10.1111/1744-7917.12891

[39] C. L. Kwapich, B. Hölldobler, Destruction of spiderwebs and rescue of ensnared nestmates by a granivorous desert ant (*Veromessor pergandei*). Am. Nat. **194**, 395–404 (2019). 31553216 10.1086/704338

[40] G. Li, X. Zou, C. Lei, Q. Huang, Antipredator behavior produced by heterosexual and homosexual tandem running in the termite *Reticulitermes chinensis* (Isoptera: Rhinotermitidae). Sociobiology **60**, 198–203 (2013).

[41] K. Matsuura, E. Kuno, T. Nishida, Homosexual tandem running as selfish herd in *Reticulitermes speratus*: Novel antipredatory behavior in termites. J. Theoret. Biol. **214**, 63–70 (2002). 11786032 10.1006/jtbi.2001.2447

[42] Z. Zhao, X. Yin, C. Shih, T. Gao, D. Ren, Termite colonies from mid-Cretaceous Myanmar demonstrate their early eusocial lifestyle in damp wood. Natl. Sci. Rev. **7**, 381–390 (2020). 34692054 10.1093/nsr/nwz141 PMC8288961

[43] T. Chouvenc , Revisiting *Coptotermes* (Isoptera: Rhinotermitidae): A global taxonomic road map for species validity and distribution of an economically important subterranean termite genus. Syst. Entomol. **41**, 299–306 (2016).

[44] T. M. Therneau, coxme: Mixed effects Cox models, Version 2.2-18. cran.r-project.org/package=coxme. Accessed 17 October 2022.

[45] N. Mizumoto, T. Bourguignon, Light alters activity but does not disturb tandem coordination of termite mating pairs. Ecol. Entomol. **48**, 145–153 (2022).

[46] O. Yamanaka, R. Takeuchi, UMATracker: An intuitive image-based tracking platform. J. Exp. Biol. **221**, 1–24 (2018). 10.1242/jeb.182469 29954834

[47] A. Mathis , DeepLabCut: Markerless pose estimation of user-defined body parts with deep learning. Nat. Neurosci. **21**, 1281–1289 (2018). 30127430 10.1038/s41593-018-0209-y

[48] T. Nath , Using DeepLabCut for 3D markerless pose estimation across species and behaviors. Nat. Protoc. **14**, 2152–2176 (2019). 31227823 10.1038/s41596-019-0176-0

[49] K. He, X. Zhang, S. Ren, J. Sun, Deep residual learning for image recognition. arXiv [Preprint] (2015). 10.48550/arXiv.1512.03385 (Accessed 14 September 2022).

[50] E. Insafutdinov, L. Pishchulin, B. Andres, M. Andriluka, B. Schiele, DeeperCut: A deeper, stronger, and faster multi-person pose estimation model. arXiv [Preprint] (2016). 10.48550/arXiv.1605.03170 (Accessed 14 September 2022).

[51] J. Lauer , Multi-animal pose estimation, identification and tracking with DeepLabCut. Nat. Methods **19**, 496–504 (2022). 35414125 10.1038/s41592-022-01443-0 PMC9007739

[52] J. Lever, M. Krzywinski, N. Altman, Logistic regression. Nat. Methods **13**, 541–542 (2016).

[53] R Core Team, R: A language and environment for statistical computing (R Foundation for Statistical Computing, Vienna, Austria, 2023). https://www.R-project.org. Accessed 10 February 2024.

[54] N. Mizumoto, tandem-fossil. Github. https://github.com/nobuaki-mzmt/tandem-fossil. Deposited 7 December 2022.

[55] N. Mizumoto, S. Hellemans, M. Engel, T. Bourguignon, A. Bucek, nobuaki-mzmt/tandem-fossil: Version 1.0.1. Zenodo. 10.5281/zenodo.10557251. Deposited 23 January 2024.

