# Supplementary material for: Extinct and extant termites reveal the fidelity of behavior fossilization in amber
Source: Proc Natl Acad Sci U S A. 2024 Mar 5;121(12):e2308922121. doi: 10.1073/pnas.2308922121 (PMC10963005; doi:10.1073/pnas.2308922121)
Supplement: Supplementary file 1 — Appendix 01 (PDF) [file pnas.2308922121.sapp.pdf]

PNAS

Supporting Information for
Extinct and extant termites reveal the fidelity of behavior fossilization in amber

Nobuaki Mizumoto, Simon Hellemans, Michael S Engel, Thomas Bourguignon, Aleš Buček

Nobuaki Mizumoto
Email: nobuaki.mzmt@gmail.com
Aleš Buček
Email: bucek.ales@gmail.com

This PDF file includes:

Figures S1 to S6
Tables S1
Legend for Video S1 to S3

Other supporting materials for this manuscript include the following:

Video S1 to S3

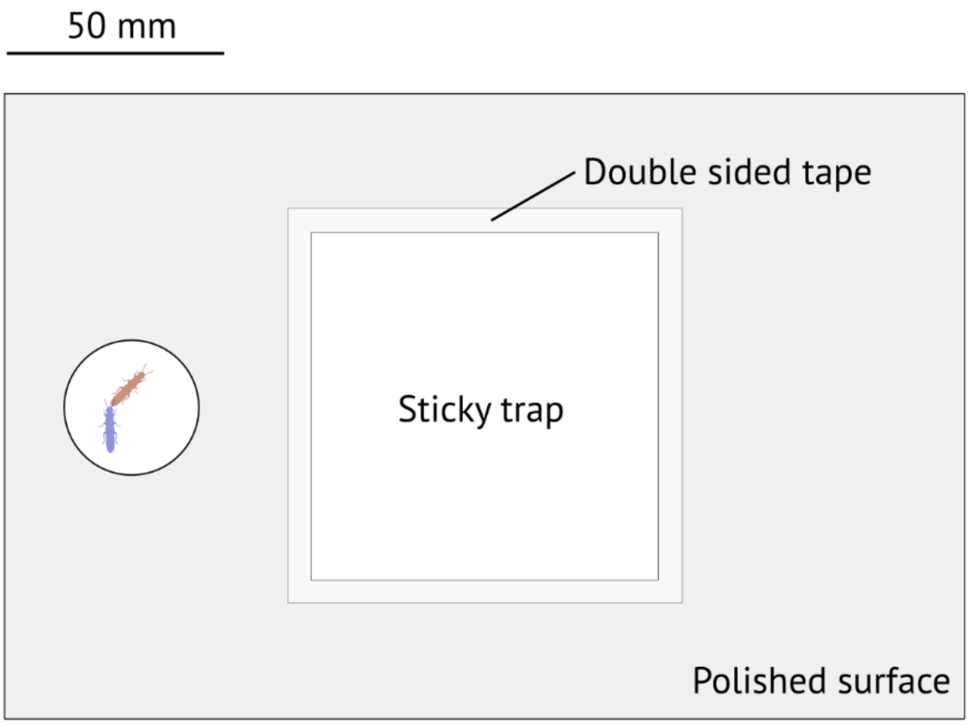

Figure S1. Experimental setup for observing tandem pair behavior on sticky surface simulating resin. A pair was isolated below a Petri dish (Φ=40mm) until a tandem run was initiated.

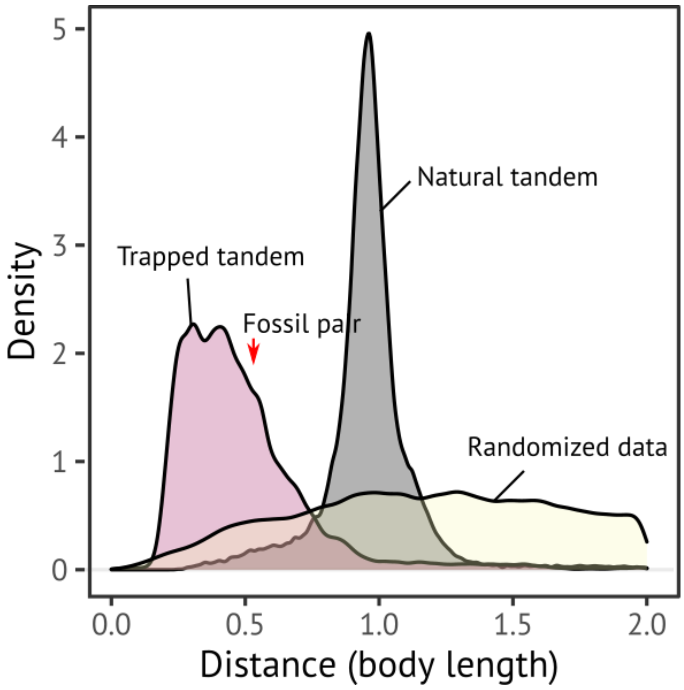

Figure S2. Distribution of distances between centroid of females and males across different contexts of tandem runs. The red arrow indicates the female-male distance in the amber inclusion.

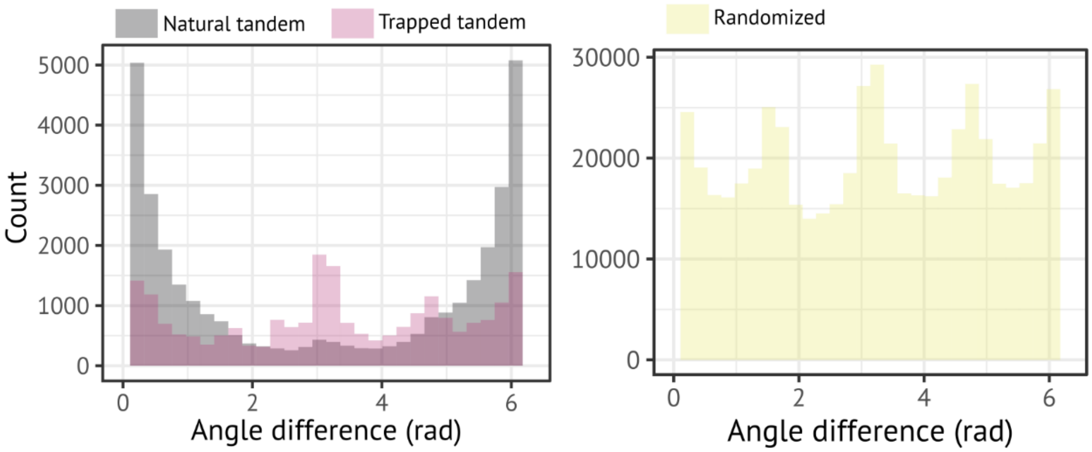

Figure S3. Distribution of the distance in heading direction between females and males across different context of tandem runs.

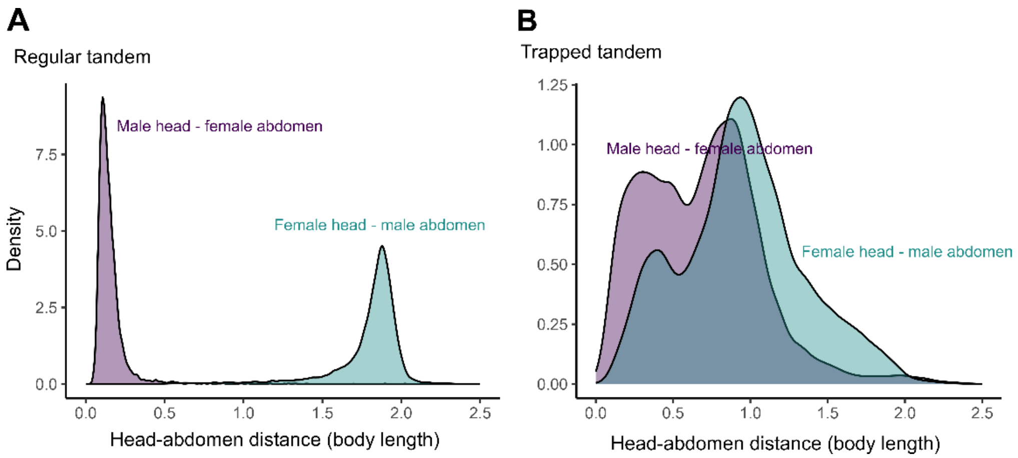

Figure S4. Density distribution of the distance between head and abdomen tip in (A) regular tandem runs and (B) trapped tandem pairs. In regular tandems, fHead-mTip distance is larger than fTip-mHead distance in 99.6% of time, while in trapped tandem, fHead-mTip distance was larger than fTip-mHead distance only 60.9% of time.

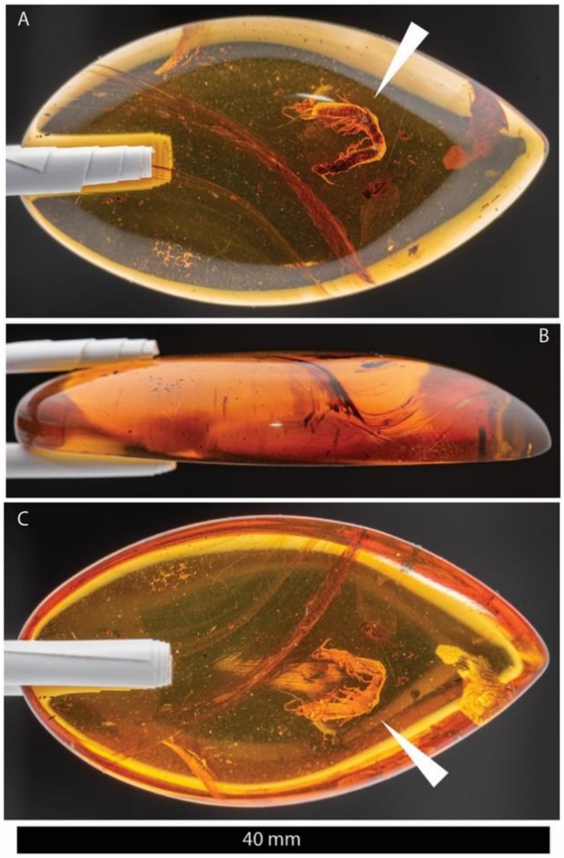

Figure S5. The fossil tandem NMP T3532. (A) Dorsal side of the termite tandem; (B) sagittal view; and (C) ventral side of the termite tandem. White arrowheads indicate the position of the termite tandem.

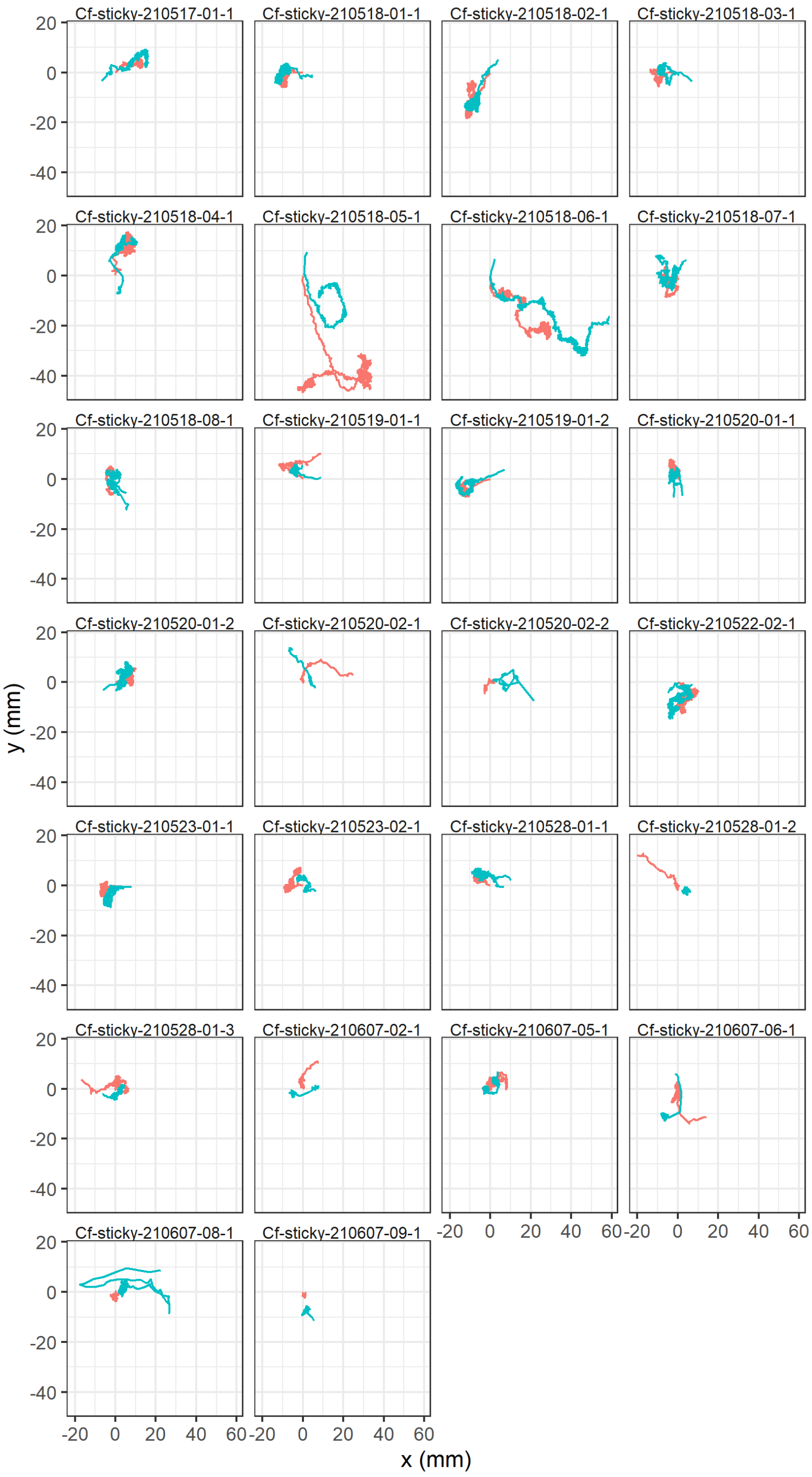

Figure S6. Trajectories of all pairs that entered the sticky trap area.

Table S1. Overview of scanning parameters.

ScanID	Description	Voxel size (μm)	Source setting	Optical magnification	Source distance; detector distance (mm)	Exposure time (s)
fossil2b	abdominal tip of male and head of female	3.45	40kV, 75μA	4x	27; 26	15
fossil2c	abdominal tip of female	2.17	40kV, 75μA	4x	26;55	17
fossil2e	both termite individuals	9.46	40kV, 75μA	0.4x	22;137	18

Supplementary Video S1 (separate file). Rotating view of a reconstructed microtomographic section of the fossil amber piece. Termites are highlighted in orange. The amber matrix is visualized as a volume using a range of grey intensities to distinguish subtle differences in material densities. Resin flow boundaries are visible as faint undulating planes.

Supplementary Video S2 (separate file). Animated slicing view through the reconstructed microtomographic section of the fossil amber piece. The area corresponding to the termites is highlighted in orange on each slice. The amber matrix is visualized as a volume using a range of grey intensities to distinguish subtle differences in material densities. Resin flow boundaries are visible as faint undulating lines.

Supplementary Video S3 (separate file). Example of a termite mating pair caught by a sticky trap.
